# Case Report: Transthyretin Glu54Leu—a rare mutation with predominant cardiac phenotype

**DOI:** 10.3389/fcvm.2023.1228410

**Published:** 2023-10-31

**Authors:** Mariana Gospodinova, Sashka Zhelyazkova, Teodora Chamova, Ognyan Asenov, Zornitsa Pavlova, Tihomir Todorov, Dilyana Mikova, Yordan Palashev, Ivan Gruev, Atanas Kundurdjiev, Albena Todorova, Ivailo Tournev

**Affiliations:** ^1^ Expert Centre for ATTR Cardiac Amyloidosis, St Ivan Rilski University Hospital, Sofia, Bulgaria; ^2^ Clinic of Neurology, Aleksandrovska University Hospital, Medical University, Sofia, Bulgaria; ^3^ Genetic Medico-Diagnostic Laboratory “Genica”, Sofia, Bulgaria; ^4^ Department of Nuclear Medicine, St Ivan Rilski University Hospital, Sofia, Bulgaria; ^5^ Clinical Center of Nuclear Medicine and Radiology, Medical University, Sofia, Bulgaria; ^6^ National Multi-profile Transport Hospital “Tsar Boris III”, Sofia, Bulgaria; ^7^ Clinic of Nephrology, St Ivan Rilski University Hospital, Sofia, Bulgaria; ^8^ Department of Medical Chemistry and Biochemistry, Medical University Sofia, Sofia, Bulgaria; ^9^ Department of Cognitive Science and Psychology, New Bulgarian University, Sofia, Bulgaria

**Keywords:** amyloidosis, transthyretin, p.Glu74Leu (Glu54Leu) mutation, cardiomyopathy, polyneuropathy

## Abstract

We report two unrelated Bulgarian families with hereditary transthyretin (ATTR) amyloidosis due to a rare p.Glu74Leu (Glu54Leu) pathogenic variant found in seven individuals—three of them symptomatic. Only one family with the same variant and with a Swedish origin has been clinically described so far. Our patients are characterized by predominant cardiac involvement, very much similar to the Swedish patients. Although the initial complaint was bilateral carpal tunnel syndrome, advanced amyloid cardiomyopathy was found in two symptomatic carriers at diagnosis with heart failure manifestations. The neurological involvement was considered as mild, with mainly sensory signs and symptoms being present. We followed a non-biopsy algorithm to confirm the diagnosis. Tafamidis 61 mg has been initiated as the only approved disease modifying treatment for ATTR cardiomyopathy. Clinical stability in the absence of adverse events has been observed at follow up.

## Introduction

Hereditary transthyretin amyloidosis (ATTRv) is an autosomal dominant disease caused by more than 130 mutations in the transthyretin (*TTR)* gene (1, 2). The disorder can be classified in two main phenotypes: cardiac and neurological, which are rarely found in an isolated form (3, 4). Bulgaria is among the countries with a high number of ATTRv amyloidosis cases. The most common *TTR* pathogenic variant in our country is p.Glu109Gln (Glu89Gln), followed by four other less prevalent mutations—p.Val50Met (Val30Met), p.Ser97Phe (Ser77Phe), p.Gly67Glu (Gly47Glu), and p.Ser72Pro (Ser52Pro) (5–9). Patients diagnosed with these variants are characterized by a mixed phenotype—neurological and cardiac.

Here, we report two unrelated families with ATTRv amyloidosis, carriers of the sixth mutation discovered in Bulgaria—p.Glu74Leu (Glu54Leu) with predominant cardiac manifestations. This variant is new for our country and very rare worldwide.

## Methods

Diagnostic evaluation started with clinical and family history, followed by physical examination, electrocardiogram (ECG), and blood and biochemistry tests. Cardiac evaluation consisted of 2D echocardiography, tissue Doppler imaging, and speckle tracking echocardiography. The final diagnosis of transthyretin amyloid cardiomyopathy (ATTR-CM) was determined using a non-biopsy algorithm, through ^99m^Tc-pyrophosphate (^99m^Tc-РYP) bone scintigraphy with additional SPECT/CT imaging. Electrophoresis with immunofixation of serum and urine and serum-free light chains were performed to rule out light chain amyloidosis (AL). According to the Perugini grading scale, grade 2 and 3 bone tracer myocardial uptake in the absence of monoclonal gammopathy confirmed the diagnosis (10).

A comprehensive neurological assessment was performed to evaluate neurological involvement. Neurophysiological tests encompassed nerve conduction studies (NCS), sympathetic skin response (SSR), and electrochemical skin conductance (ESC), measured using Sudoscan (11). Motor (tibial, peroneal, median, and ulnar) and sensory (sural, superficial peroneal, median, and ulnar) nerves were assessed on both body sides (12).

Testing for possible amyloidogenic mutations in the *TTR* gene was performed using a standard Sanger sequencing procedure for the four *TTR* exons. The sequencing reaction was performed using a BigDye Terminator Cycle Sequencing Kit v.3.1. The sequenced fragments were analyzed on an ABI3130 Genetic Analyzer using Sequencing Analysis v.5.1.1 software.

Two of the patients have been followed up in terms of cardiac and neurologic involvement for a period of 12 months and one for 6 months.

## Clinical report

### Family 1

The proband was a 55-year-old male, diagnosed with bilateral carpal tunnel syndrome at the age of 43, who subsequently underwent surgery of both hands 6 years later. At the age of 53, the patient was admitted to a neurological department with ischemic stroke, presenting with sensory-motor aphasia. No prior complaints consistent with peripheral or autonomic nervous system involvement, such as paresthesia and dizziness, were reported. Although he had no prior history of cardiovascular disease, he underwent cardiac evaluation because of subsequent manifestation of some cardiac symptoms, including chest discomfort, palpitations, shortness of breath, fatigue on exertion, and edema of both legs. Echocardiography revealed left ventricular hypertrophy and reduced ejection fraction. Holter ECG was performed, and paroxysmal atrial fibrillation was registered, indicating the cardioembolic origin of the stroke. Anticoagulation with 20 mg Rivaroxaban was initiated and amiodarone was also prescribed as an antiarrhythmic drug. Five months later, the patient was admitted to a cardiology department due to heart failure manifestations. The coronary angiography was normal. Because of echocardiography findings of significant left ventricular hypertrophy, the patient was referred to a tertiary center for diagnostic evaluation of cardiomyopathy with a hypertrophic phenotype. Cardiac magnetic resonance imaging showed hypertrophy of both ventricles and delayed subendocardial gadolinium enhancement, consistent with amyloidosis.

### Physical examination

On initial physical examination, the patient had a blood pressure of 125/93 mmHg, a heart rate of 62 beats/min with regular rhythm, no audible murmur, and rales on auscultation. There were signs of elevated jugular venous pressure, some tenderness in the liver, and edema of both lower extremities below the knees.

The family history revealed that his mother died at the age of 68 due to heart failure and that four other deceased members from two previous generations could also have been possibly affected because of the history of heart disease.

### Cardiac evaluation

**Figure 1:**
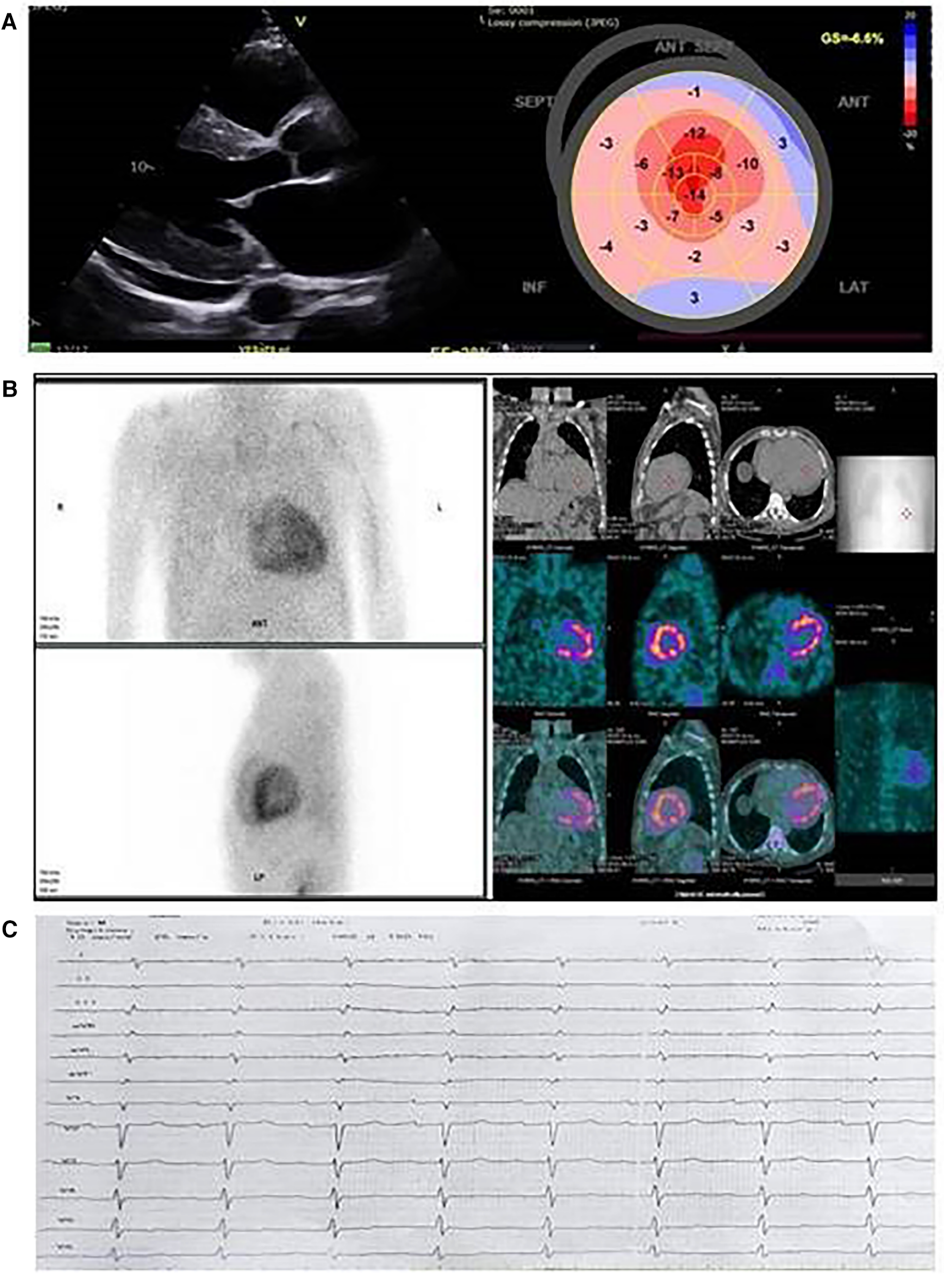
Cardiac imaging of the proband from family 1. (**A**) Echocardiographic images; left, parasternal long axis view: thick left ventricular walls with sparkling appearance, small pericardial effusion, and thickened mitral valve leaflets; right, speckle tracking echocardiography and reduced global longitudinal strain with relative apical sparing in the bull's eye image. (**B**) ^99m^Tc-pyrophosphate (^99m^Tc-РYP) bone scintigraphy with SPECT/CT images. ^99m^Tc-РYP myocardial uptake was assessed as grade 3. (**C**) Electrocardiography: sinus rhythm, low voltage, right axis deviation, and A-V block I^st^ degree.

Echocardiography showed a significantly increased left ventricular wall thickness of approximately 19 mm, with a reduced ejection fraction (EF) of 28%, a global longitudinal strain (GLS) of −6.6%, stage III diastolic dysfunction, thickened valve leaflets, and small pericardial effusion (Figure 1A). As a diagnosis of cardiac amyloidosis was highly likely, the most important next step was to schedule 99TcPYP bone scintigraphy and to rule out AL amyloidosis. The free light chains kappa and lambda values and their ratio were within the reference range, and no monoclonal protein was detected on immunofixation. Routine blood chemistry and urinary analysis were unremarkable. NTproBNP was significantly elevated: 11,630 pg/ml (Table 1). The glomerular filtration rate (GFR) was 79 ml/min/1.73 m^2^. The ^99m^Tc–PYP bone scintigraphy and the subsequent SPECT/CT showed a H/CL ratio of 2.3 on the planar images and a grade 3 visual score on the SPECT/CT images, with myocardial uptake greater than rib uptake. These findings together ruled out AL amyloidosis and confirmed the diagnosis of ATTR-CM (Figure 1B). The ECG revealed sinus rhythm, first-degree atrioventricular block, and low voltage in the peripheral leads (Figure 1C). As variant ATTR amyloidosis was highly likely because of the relatively young age of the patient, we performed a comprehensive neurological evaluation and genetic testing.

**Table 1:** Clinical characteristics at baseline and at follow up of patients diagnosed with ATTR amyloidosis caused by the p.Glu74Leu variant.

Patient number	1		2		3	
Age at disease onset (years)	43		52		45	
Age at initial evaluation (years)	57		54		57	
Follow up	Baseline	At 12 months	Baseline	At 12 months	Baseline	At 6 months
NYHA class	III	III	II	II	III	II
Heart arrhythmia/conduction disturbances	AF, A-V block 1st degree	AF, A-V block 1st degree	No	No	A-V block 1st degree	A-V block 1st degree
Polyneuropathy stage	Stage I	Stage I	Stage I	Stage I	Stage I	Stage I
Abnormal HRV^a^/Symptoms of Autonomic neuropathy	Yes	Yes	Yes	Yes	Yes	Yes
Carpal tunnel syndrome	Yes	Yes	No	No	Yes	Yes
Low voltage ECG	Yes	Yes	No	No	Yes	Yes
IVSD (mm)	19	18	13	13	19	19
LVEDD (mm)	52	53	44	48	53	51
RWT	0.73	0.68	0.59	0.54	0.75	0.75
LVEF (%)	38	37	63	69	45	45
RF	Yes	Yes	No	No	Yes	Yes
GLS (%)	−6.6%	−6.2%	−18.6	−18.4	−9.0%	−9.2%
NTpro-BNP (ng/L)	11,630	10,066	104	105	2,951	2,669
GFR (ml/min/1.73 m^2^)	79	70	68	66	67	54
NAC stage	Stage II	Stage II	Stage I	Stage I	Stage I	Stage I

^a^Heart rate variability.

### Neurological evaluation

The neurological examination was difficult to assess due to sensory and motor aphasia. Muscle strength and ambulation were preserved. Tendon reflexes were brisk on the right. Plantar reflexes were bilaterally attenuated. Sensation testing was not possible due to impaired verbal communication. NCS during the initial evaluation were consistent with bilateral carpal tunnel syndrome, mild axonal sensory and motor polyneuropathy, with absent SNAPs in the lower limbs, and a decreased amplitude of SNAPs for the median nerves. The amplitude of CMAP was decreased for both peroneal nerves. Sudoscan was normal, while a sympathetic skin response test showed no response after electric stimulation in both hands and feet.

### Genetic testing for ATTRv

**Figure 2:**
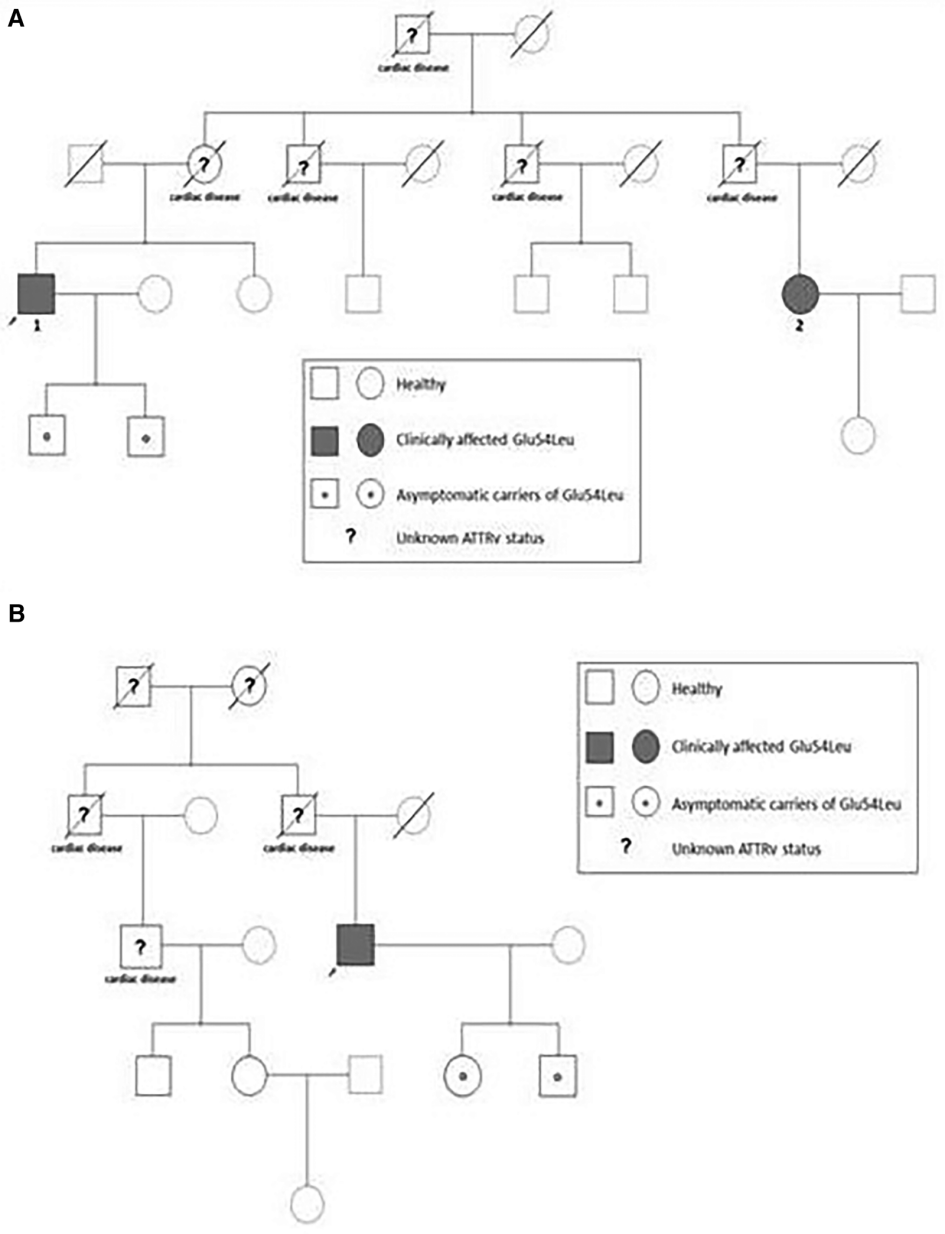
Family pedigree. Relationship of the affected individuals in the families with the p.Glu74Leu variant. Arabic numerals refer to the case numbers in the text. (**A**) Family 1. (**B**) Family 2.

Genetic testing for *TTR* mutations in the proband revealed a heterozygous *TTR* variant c. 20_221delGAinsCT. This is a complex genetic variant representing a substitution of two adjacent nucleotides in exon 3 of the *TTR* gene. Segregation analysis of the family proved that the two nucleotide substitutions are located on the same allele and lead to a change of the negatively charged amino acid glutamate, with the neutral amino acid leucine at position 74 of the amino acid sequence of the transthyretin protein [TTR:c.220_221delGAinsCT, (p.Glu74Leu)]. This genetic variant, known as p.Glu74Leu, is classified as pathogenic according to the genetic variant classification recommendations of the American College of Medical Genetics and Genomics (13), evidence categories PM1, PM5, PP5, and PM2. These results confirmed a diagnosis of hereditary transthyretin amyloidosis in the proband. Genetic testing of the relatives at risk was recommended. His two sons, aged 25 and 29, were found to be asymptomatic. The patient has four cousins, three males and one female; all were tested, and the female was diagnosed as a carrier of the mutation (Figure 2A).

### Treatment and follow up

Following the guidelines, Tafamidis 61 mg has been initiated for ATTR-CM with heart failure and stage I polyneuropathy (14). Treatment with torasemide, eplerenone, and rivaroxaban has also been prescribed. On the fifth month, the patient was admitted to hospital with signs and symptoms of heart failure—breathlessness and edema of the legs, with clinical improvement after intravenous application of loop diuretic. SGLT2 inhibitor has been added to the therapy. In the following months, the patient was stable without heart failure exacerbations and his wife reported an improvement in the quality of life. The results from the follow up visit at 12 months are presented in Table 1. New rhythm and conduction disturbances were not registered on Holter ECG. No adverse events related to Tafamidis have been observed. The NCS parameters remained stable for the follow up period.

The second patient, a 54-year-old female cousin of the proband, was diagnosed with p.Glu74Leu mutation through family screening. She had a history of arterial hypertension, autoimmune thyroiditis with hypothyroidism, obesity, combined glucose intolerance, and adenoma of the left adrenal gland with no clinical significance. She was taking Lisinopril 5 mg and l-tyroxin 50 µg. At the age of 52, she started having generalized fatigue and shortness of breath with physical activity. At the age of 54, a pain in the thumbs appeared, followed by numbness and paresthesia in both hands. She also described early satiety.

### Cardiac evaluation

ECG revealed sinus rhythm, left axis, and no pathological changes. Echocardiography revealed a moderately increased left ventricular wall thickness of approximately 13 mm, with a preserved EF and GLS of −18.4%, and grade I diastolic dysfunction. On 24 h Holter ECG, no significant rhythm or conduction disturbances were registered. A H/CL ratio of 2.2 was found on the ^99m^Tc-PYP bone scintigraphy and grade 3 myocardial uptake was confirmed on the subsequent SPECT/CT. Her blood tests (complete blood count, liver enzymes, urea, creatinine, and TSH) and urine sediment were within normal ranges. The blood glucose, hemoglobin A1C, and uric acid levels were slightly elevated. NT proBNP evaluated at the time of diagnosis was 104 pg/ml (Table 1).

### Neurological evaluation

The patient had neither muscle atrophy nor weakness. Deep tendon reflexes were attenuated in four limbs. Pain and temperature sensations were decreased bilaterally below the knees and in the hands. Vibration sense was slightly decreased in the big toes (6/8); however, the position sense was preserved. NCS were consistent with axonal sensory polyneuropathy in the lower limbs, with absent SNAPs on the sural and superficial peroneal nerves. Sudoscan was normal. A sympathetic skin response test showed low amplitudes in the upper extremities and normal amplitudes and latencies in the lower extremities. Her FAP stage and PND score were 1.

Treatment with Tafamidis was initiated. We have been following the patient for 12 months, with no signs of heart failure exacerbations or rhythm disturbances (Table 1). The mild neurological involvement remained stable.

### Family 2

The patient was a 57-year-old male with clinical onset of carpal tunnel syndrome at the age of 45. The disease initially affected the right hand, and surgery was performed the same year. In 2014, the left hand was also affected, and the patient underwent a second surgery. In the years after, he complained only of intermittent tingling in both hands. Since the age of 54, the patient started to experience fatigue on exertion and was subsequently diagnosed with heart failure. The patient had a 20 year history of arterial hypertension and hypercholesterolemia. In January 2020, the patient was admitted to a cardiology department for further evaluation and treatment. Coronary angiography did not reveal coronary artery disease. On echocardiography, left ventricular hypertrophy of 20 mm for the septum and 15 mm for the posterior wall as well as a reduced EF of 37% were described. The patient was stable for approximately 1 year; however, since January 2022, his condition became worse, with episodes of shortness of breath, edema of both legs, and generalized fatigue even after rest. In December 2022, he was admitted to a cardiology department with HF exacerbation, where he was treated with intravenous diuretics. The diagnosis at discharge was hypertrophic non-obstructive cardiomyopathy, with the following therapy: furosemide 40 mg, eplerenon 50 mg, empagliflozin 10 mg, and sacubitrl/valsartan 24/26 mg. In the discussion, cardiac amyloidosis was suspected, and the patient was referred to the amyloidosis center.

### Physical exam

His blood pressure was 115/76, heart rate 72 beats/min with regular rhythm, and no audible murmurs or rales on auscultation. Since 2022, he has also reported paresthesia in the feet.

Regarding the family history, the patient's father and uncle died at the ages of 47 and 63, respectively, with a history of heart failure. They may possibly have been affected, and his cousin is suffering from heart failure but has not yet been tested.

### Cardiac evaluation

**Figure 3:**
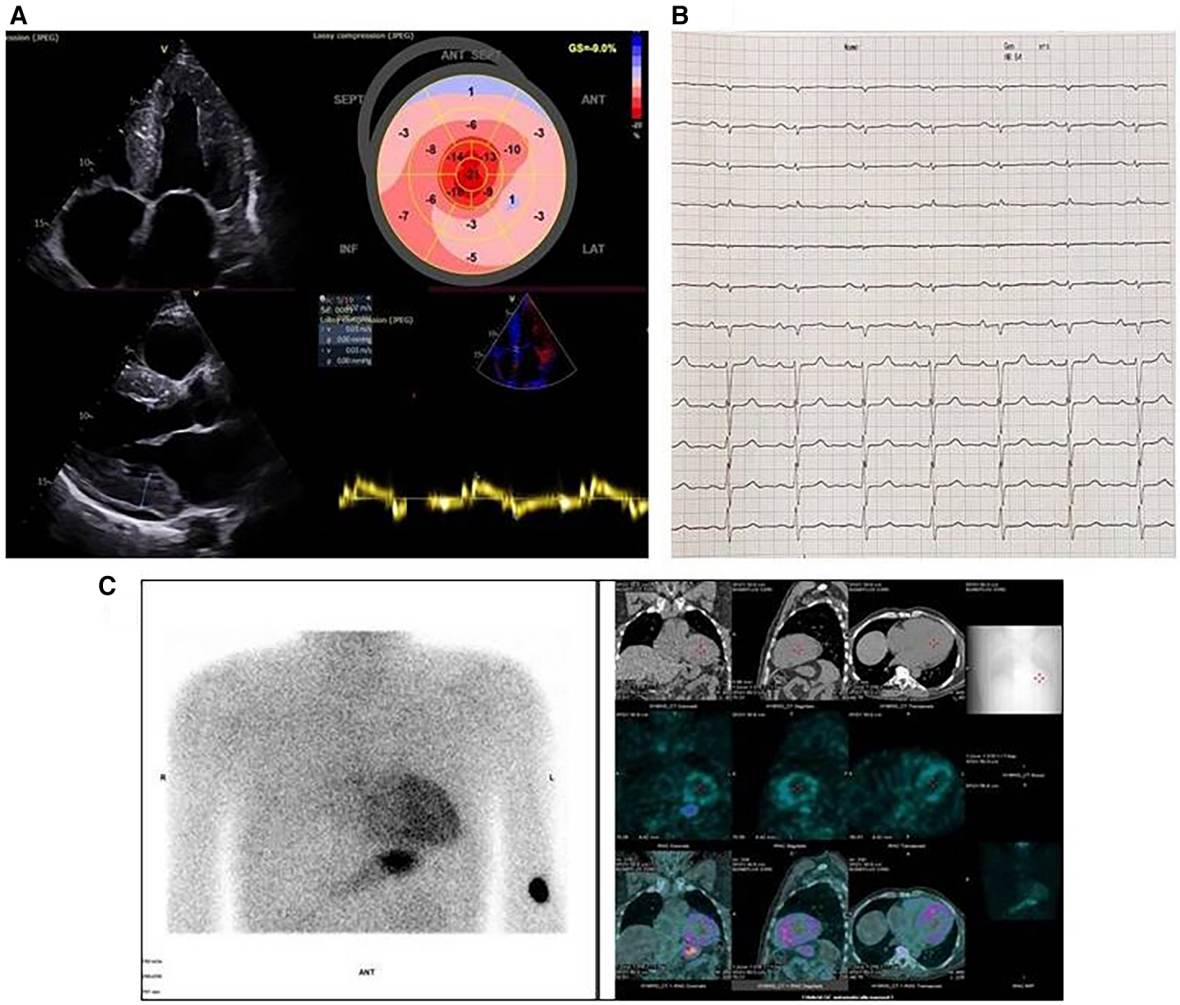
Cardiac imaging of the proband from family 2. (**A**) Echocardiographic images; upper left, parasternal apical view: thick walls, nondilated ventricles, and dilated atria; upper right, speckle tracking echocardiography: reduced global longitudinal strain with relative apical sparing on the bull's eye image; lower left, thick left ventricular walls with sparkling appearance, small pericardial effusion, and thickened mitral valve leaflets; lower right, reduced septal mitral annular tissue Doppler myocardial velocities. (**B**) Electrocardiography: sinus rhythm, low voltage, right axis deviation, and A-V block I^st^ degree. (**C**) ^99m^Tc-pyrophosphate (^99m^Tc-РYP) bone scintigraphy with SPECT/CT images. ^99m^Tc-РYP myocardial uptake was assessed as grade 3.

ECG revealed sinus rhythm and A-V block I^st^ degree (Figure 3B). Echocardiography showed a significantly increased left ventricular wall thickness of 20 mm for the septum and 19 mm for the posterior wall, reduced EF and GLS of 45% and −9.0%, respectively, stage III diastolic dysfunction, thickened valve leaflets, and small pericardial effusion (Figure 3A). 48 h Holter ECG was performed, with no registration of atrial fibrillation or conduction disturbances. ^99m^Tc–PYP bone scintigraphy was conducted, showing a H/CL ratio of 2 and grade 3 myocardial uptake on the SPECT/CT, confirming a higher myocardial uptake of the bone tracer (Figure 3C). AL cardiomyopathy was ruled out; therefore, ATTR-CM was diagnosed. His blood tests (complete blood count, liver enzymes, urea, and blood glucose) and urine sediment test yielded normal results. The GFR was 67 ml/min/1.73 m^2^. NT proBNP evaluated at the time of diagnosis was 2,951 pg/ml (Table 1).

### Neurological evaluation

No muscle atrophy or weakness were present. Plantar and brachioradial reflexes were bilaterally attenuated. Pain and temperature sensations were decreased bilaterally below the knees and in the hands. Vibration sense was decreased in lower limbs (under the level of crista iliaca), while position sense was preserved. He had bilateral carpal tunnel syndrome. NCS revealed axonal sensory polyneuropathy in the four limbs and bilateral carpal tunnel syndrome. Sudoscan was normal. A sympathetic skin response test showed no responses after electric stimulation in both hands and feet.

### Genetic testing for ATTRv

Genetic testing for *TTR* mutations in the proband revealed a heterozygous *TTR* variant c.220_221delGAinsCT, p.Glu74Leu. The daughter and son of the patient have been diagnosed as asymptomatic carriers of the mutation (Figure 2B).

Treatment with Tafamidis 61 mg has been initiated, together with furosemide, eplerenon, and empagliflozin. We have been following the patients for 6 months, with no reported adverse events (Table 1). No significant progression was observed in the NCS parameters.

## Discussion

p.Glu74Leu is a rare variant, reported in ATTRv patients from Sweden, Belgium, the UK, and Japan (15–18). The only family with a thorough clinical description so far is a Swedish family. We have identified two unrelated families with this variant, with three affected patients: two males and one female as well as four asymptomatic carriers. A common finding between the Bulgarian and Swedish patients was predominant cardiac involvement, with significantly increased LV wall thickness, restrictive filling, and reduced EF and GLS in patients at an advanced stage of the disease as well as HF signs and symptoms and atrial fibrillation. The patients diagnosed through family screening demonstrated less severe cardiomyopathy. In most patients from both countries, the age of onset was in the fourth decade, presenting with carpal tunnel syndrome prior to cardiac symptoms usually appearing in the fifth decade (15). The age of onset is similar in patients with p.Glu109Gln, the most common mutation in Bulgaria, which is also found in Italy, and in p.Glu74Gln, the most common mutation in Romania, both characterized by significant cardiac involvement (6, 8, 19). In both the Swedish and Bulgarian patients with p.Glu74Leu, mild neurological involvement and mainly sensory signs and symptoms were present. This finding differs from patients with p.Glu109Gln and p.Glu74Gln, who have infiltrative cardiomyopathy but also significant peripheral neuropathy and autonomic dysfunction (6, 19). The age of onset in p.Glu74Leu is earlier than that in patients with the most common cardiac variant p.Val142Ile, found in approximately 4% of the Afro-American population in the United States, and also than that of wild type ATTR-CM (20). Despite the similarities, heterogeneity in the age of onset and the clinical manifestations was found in both the Swedish and Bulgarian patients, also seen in other mutations (20). Our female patient demonstrated later and less severe clinical manifestations than the male patients, which is also described in other variants (6, 8). Broad intra and interfamilial variations in terms of age of onset and severity have been observed in ATTRv amyloidosis, caused by diverse mutations. Therefore, we can speculate that genetic modifiers and epigenetic factors are causing such variability (21, 22). Future research should be conducted to reach more definitive conclusions. Another similarity between the Bulgarian and Swedish patients with p.Glu74Leu is that they originate from endemic regions with the most common mutations in each country: p.Glu109Gln in Bulgaria and p.Val50Met in Sweden (6, 15).

The Swedish team described a different amyloid fibril composition in these patients, with possible differences in fibril structure as well as the mechanism of fibrillogenesis, compared to the already known A and B types of fibrils. Moreover, they hypothesized that patients with p.Glu74Leu mutations could be less likely to benefit from TTR stabilization by small molecules such as Diflunisal and Tafamidis because of the locus of the mutation; treatment with patisiran or inotersen could also be an option for these patients (15).

In our study, heart failure manifestations were stabilized and an improvement in the self-reported quality of life was observed during the follow up. No adverse events were reported related to the Tafamidis treatment. No significant changes in the echocardiographic parameters or in the NAC stage, with a slight decrease in NT-proBNP levels, were found. New rhythm and conduction disorders were not registered. No significant progression in the NCS parameters was observed.

The affected genetic locus at codon 74 of the TTR protein coding sequence could be a hot spot for accumulation of amyloidogenic variants, with at least six pathogenic variants currently reported (23–25). A founder pathogenic variant has been identified in Romanian patients: TTR:c.220G >C, p.Glu74Gln (19). The p.Glu74Gln variant represents a substitution of a negatively charged amino acid with a polar amino acid residue; these are physicochemically more similar than the change of glutamate with leucine (leucine has a non-polar amino acid side chain). Additional comparative functional proteomic analyses are needed to come to a proper conclusion regarding the differences of stability and pathogenicity of the two variants p.Glu74Leu and p.Glu74Gln. p.Glu74Leu occurs in two genetic forms, which, due to the degeneracy of the genetic code, result in the same amino acid substitution (c.220_221delGAinsCT and c.220_221delGAinsTT). The c.220_221delGAinsTT variant has been reported once in a patient from Belgium and once in a patient from Scotland (17). Of particular importance here is the correct nomenclatural reporting of newly discovered genetic variants in scientific publications and databases; so that genetic and phenotypic follow-up of patient populations with the same genetic variant is possible.

In conclusion, we have reported the clinical features and pedigrees of three patients, from two unrelated families, with hereditary ATTR amyloidosis with the rare p.Glu74Leu pathogenic variant. They are characterized by a predominant cardiac phenotype, presenting with progressive cardiomyopathy, heart failure signs and symptoms, and rhythm disturbances. The neurologic involvement was relatively mild, consisting of bilateral carpal tunnel syndrome and axonal polyneuropathy.

## Data Availability

The raw data supporting the conclusions of this manuscript will be made available by the authors, without undue reservation, to any qualified researcher.
